# Rural-to-Urban Migration: Socioeconomic Status But Not Acculturation was Associated with Overweight/Obesity Risk

**DOI:** 10.1007/s10903-015-0234-9

**Published:** 2015-06-19

**Authors:** Angela Hilmers, Antonio Bernabé-Ortiz, Robert H. Gilman, Ann Y. McDermott, Liam Smeeth, J. Jaime Miranda

**Affiliations:** Department of International Health, Johns Hopkins Bloomberg School of Public Health, Baltimore, MD USA; CRONICAS Center of Excellence in Chronic Diseases, Universidad Peruana Cayetano Heredia, Lima, Peru; Epidemiology Unit, School of Public Health and Administration, Universidad Peruana Cayetano Heredia, Lima, Peru; Área de Investigación y Desarrollo, A.B. PRISMA, Lima, Peru; Faculty of Epidemiology and Population Health, London School of Hygiene and Tropical Medicine, London, UK; Department of Medicine, School of Medicine, Universidad Peruana Cayetano Heredia, Lima, Peru; 5539 Carew St., Houston, TX 77096 USA

**Keywords:** Migration, Acculturation, Socioeconomic status, Physical activity, Latin America

## Abstract

To investigate whether socioeconomic status (SES) and acculturation predict overweight/obesity risk as well as the mediating effect of physical activity (PA) in the context of internal migration. Cross-sectional study of 587 rural-to-urban migrants participating in the PERU MIGRANT study. Analyses were conducted using logistic regression and structured equation modeling. Interaction effects of SES and acculturation were tested. Models were controlled for age, gender and education. Only SES was a significant predictor of overweight/obesity risk. Lower SES decreased the odds of being overweight/obese by 51.4 %. This association did not vary by gender nor was it explained by PA. Mechanisms underlying the relationship between SES and overweight/obesity may differ depending on the geographic location and sociocultural context of the population studied. Research on internal migration and health would benefit from the development of tailored acculturation measures and the evaluation of exploratory models that include diet.

## Background

Overweight and obesity are recognized global public health problems and substantial contributors to the burden of chronic health conditions such as diabetes, cardiovascular diseases, and certain forms of cancer [1, 2]. Once perceived as a public health issue restricted to industrialized societies, rapid increases in the rates of overweight/obesity and related co-morbidities are now widely documented in low-and middle-income countries (LMIC) [3–6].

Migrants constitute a distinctive and vulnerable population that, in general and compared to non-migrants, display disadvantaged risk factor profiles and an increased prevalence of non-communicable diseases [7, 8]. Studies with adult immigrants suggest that the observed increased in overweight/obesity risk in this population can be attributed to low socioeconomic status (SES) and greater acculturation levels [9, 10]. Immigrants who live below official poverty thresholds were more likely to have suboptimal diets and to report lower levels of physical activity (PA) [11, 12].

The association between acculturation—when one culture adopts the behaviors and beliefs of another—and unhealthy dietary behaviors such as low consumption of fruits and vegetables and higher intakes of saturated fat has been consistently reported in the literature^,^ [13] but the impact of acculturation on PA is less clear [14, 15]. This may be attributed to differences in the type of measures used to assess acculturation, and the lack of adjustment of confounding variables such as SES and gender [16, 17]. Length of residence, age at migration, and second language proficiency have been previously employed as proxy acculturation measures to examine migration-related changes in lifestyle habits [18–20] However, acculturation is a complex and multidirectional phenomenon that is difficult to quantify by simple static proxy indicators [21]. Acculturation goes well beyond language use and preference; it also involves a behavioral component, i.e. attitudes and values, which can vary across life domains and contexts [22]. Gender is a largely overlooked factor that shapes both migration and acculturation. Gender variations in the process of acculturation may occur due to lifestyle differences or unique responses to social, economic and behavioral risk factors. [23–25] Furthermore, the trajectory of PA after migration is likely complex with males and females possibly displaying distinct PA patterns with increasing acculturation [14]. Finally, there is an interactive effect between SES and acculturation that should be considered when examining the impact of acculturation on health behaviors [16].

As with country-to-country migration, within country rural-to-urban migration may also be associated with the loss of traditional and protective habits, and the adoption of unhealthy behaviors that are prominent in the host environment [26, 27]. Nevertheless, its association with overweight/obesity risk remains under-researched, let alone addressed, thereby limiting opportunities to better understand immigrant health differentials and their link to health and gender disparities among the ethnic populations.

Using data from a representative sample of rural–to–urban migrants within one country, we attempt to expand our knowledge of the impact of internal migration on health as well as address some of the limitations of previous studies. We consider gender while exploring the association between sociocultural factors and overweight/obesity, improve on research that use single item measures of acculturation, and create a hypothetical model that incorporates PA.

## Methods

### Study Design

A cross–sectional study was conducted using data from PERU MIGRANT (PEru’s Rural to Urban MIGRANTs). The PERU MIGRANT study was designed to investigate differences in specific cardiovascular disease risk factors between migrant and non–migrant groups [28]. Data used in this study were limited to the migrant group participants and collected in 2007. Ethical approval was obtained from Universidad Peruana Cayetano Heredia in Peru and London School of Hygiene and Tropical Medicine in the United Kingdom. All study participants provided written informed consent.

### Subjects and Setting

Participants were rural-to-urban migrants born in Ayacucho—a rural state in the Andean region of Peru—who later migrated to and were permanently living in an urban area of Lima. Participants were a product of the mass migration phenomena that occurred since the 1980s when patterns of migration in the Andean region changed dramatically mostly due to increasing political unrest and violence [28]. This process of forced migration provides a unique opportunity to assess the impact of SES and acculturation on overweight/obesity risk in a wide variety of migrants, not only among those who “chose” to migrate. Pregnant women were excluded because of gestational weight gain, as well as individuals suffering from mental disorders as this could affect the accuracy of their responses and the completeness of the survey.

### Conceptual Model

**Fig. 1:**
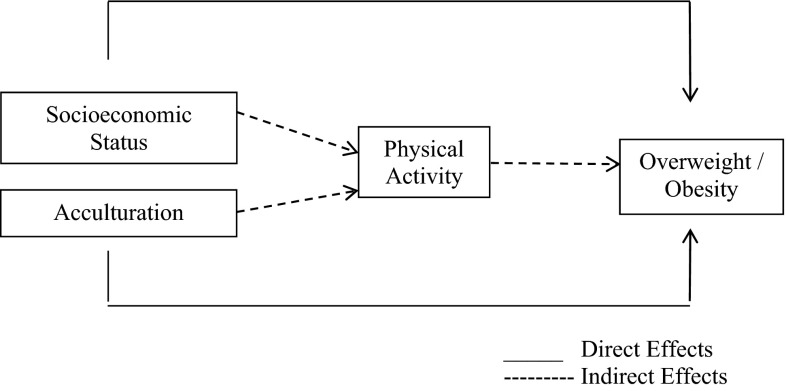
Conceptual model

A conceptual model was developed based on empirical evidence relating sociocultural influences to the dependent variable, overweight/obesity (Fig. 1) [29, 30]. The model was grounded in Bandura’s Social Cognitive Theory which postulates that a dynamic interplay of personal factors (e.g., acculturation), agent’s behavior (e.g., PA) and social environment (e.g., SES) create interactions that result in specific outcomes (e.g., overweight/obesity) [31]. SES and acculturation were hypothesized to have (1) direct effects on overweight/obesity; and (2) indirect effects on overweight/obesity through PA. In this context, PA represents an intermediary process that leads from the independent variables (SES and acculturation) to the dependent variable (overweight/obesity). An illustrative example is the higher prevalence of overweight/obesity observed in developed areas among migrants with lower SES as a result of reduced levels of PA [32, 33]. Associations between sociocultural factors and overweight/obesity were hypothesized to vary by gender based on literature addressing gender disparities related to income and cultural factors [24, 25].

## Measures

### Demographic and Socioeconomic Characteristics

Trained community field workers administered a demographic survey to all eligible participants. Information on age, gender, educational level, household income, and asset possession were collected. Place of birth and setting (rural or urban) was assessed to establish a rural–to–urban migration pattern. Rural–to–urban migration was defined as the movement of individuals from the countryside into the cities, often the metropolitan cities of a country [34]. A multi–deprivation index, a more reliable indicator of poverty than income [35], was estimated based on the aggregated number of deprivations in education (none or incomplete primary education), household income (<US$150 per month) and asset possession (lowest tertile of possessions weighted asset index) per individual. As we continue through this paper we will use the term SES to refer to an individual’s “deprivation index”. Participants showing deprivation in more than two of the given deprivation indicators were considered of low SES while those presenting none to one deprivation indicators were classified as high SES.

### Anthropometrics

Height and weight were obtained at a local clinic by trained study staff. Height was measured to the nearest 0.1 cm using a stadiometer and standard tools. Body weight with light clothing was measured to the nearest 0.05 kg using an electronic, self-calibrating digital scale (SECA 940 Model scale). Body mass index (BMI) was calculated using the formula [weight (kg)/height (m [2] )]. Overweight and obesity were defined as BMI ≥ 25 kg/m [2] and BMI ≥ 30 kg/m [2] respectively for men and women according to current accepted guidelines. [36] In this study, the overweight and obesity groups were combined to insure sufficient power and enable us to focus on all individuals at an “unhealthy weight” represented by a BMI ≥ 25 kg/m [2].

### Physical Activity

The short version of the International Physical Activity Questionnaire (IPAQ–SF) was employed to assess PA in the past 7 days. The IPAQ–SF is considered a cost–efficient method for PA activity surveillance in different populations [37] Reliability (M = 0.80) and validity (M = 0.30) correlations of this instrument are comparable to other self-report instruments [38]. Responses were converted to Metabolic Equivalent Task minutes per week (MET—min/wk) and scores were categorized into moderate–to–high and low PA. Moderate–to–high PA was defined as five or more days of any combination of walking and moderate or vigorous intensity activities achieving at least 600–3000 MET minutes per week [37]. Low PA was represented by <150 MET minutes in 1 week.

### Acculturation

Ten items were adapted from existing acculturation scales with evidence of good reliability and validity to assess [39, 40]: (1) language use and proficiency (7 items); (2) ethnic–social relations (2 items); and (3) media use (1 item). Participants were asked to name the first language they learned to speak as well as their competence and preferences for speaking Spanish or Quechua (language spoken primarily in the Andes) in their interpersonal relationships (e.g., with children, spouse and friends). Ethnic–social relations captured attitudes regarding traditional and cultural activities and the preferred ethnicity of those with whom the participant interacts. Finally, one item measured use and preference of Spanish or Quechua language media.

The acculturation items were rated in a 4–point and 5–point scales. Responses were transformed to 1 (responses favoring Spanish) or 0 (responses favoring Quechua) following the method of transformation described by Deyo et al. [39]. A continuous score of acculturation was calculated by summing all transformed item—responses. Scores ranged from 0 to 10 with higher scores indicating higher acculturation levels.

## Statistical Analysis

Student *t* test (for continuous variables) and Chi–square test (for categorical variables) were conducted to examine differences in participant characteristics by weight status. Logistic regression investigated associations between the two independent variables and overweight/obesity. All models were adjusted for age, education and PA. Because SES may be confounded with acculturation, the interactive effect between SES and acculturation was tested and the model was adjusted for SES or acculturation depending on the associations examined.

### Structural Equation Modeling (SEM)

Data were analyzed using path models, which is a special case of SEM with mean—adjusted weighted least squares estimation. The primary outcome variable for this analysis was the dichotomous variable overweight/obesity status (i.e., overweight/obese = 1 versus non–overweight/obese = 0). The estimated multi–deprivation index was dichotomized into low SES = 1 and high SES = 0. Acculturation was included as a continuous variable in the model.

SES and acculturation level were hypothesized to have direct and mediating effects on overweight/obesity through PA (Fig. 1). Solid arrows indicate a direct path while dashed arrows indicate an indirect effect. SME analyses also explored whether gender moderated the strength or direction (i.e., positive or negative) of the relationship between SES or acculturation and overweight/obesity. Analyses were conducted in Mplus (version 6.11, 1998–2011, Muthén & Muthén, Los Angeles, CA) and in Statistical Analysis Systems (version 9.3, 2011, SAS Institute Inc., Cary, NC).

## Results

### Description of Participants

A total of 587 participants were included in the analysis. The mean age of the sample was 47.8 years (SD ± 11.7); 52 % were females; and 67 % were overweight/obese (Table 1). Most participants reported engaging in moderate to high PA levels. When comparing overweight/obese to non–overweight/obese individuals, no significant differences were observed in SES, PA or acculturation levels.

**Table 1:** Baseline Characteristics of the Study Population by Weight Status

	Total (N = 587)	Non-overweight/Obese (n = 192)	Overweight/obese (n = 395)	*p* value^a^
*Age* (*mean, SD*)	47.78 (11.66)	47.27 (12.78)	47.91 (10.95)	0.55
*Gender* (*%*)				
Female	309 (52.64)	85 (44.27)	224 (56.71)	**<0.01**
Male	278 (47.36)	107 (55.73)	171 (43.29)	
*Education level* (*%*)				
None/some elementary school	181 (30.89)	54 (28.13)	127 (32.23)	**<0.001**
Elementary school/some high school	225 (38.40)	63 (32.81)	162 (41.12)	
High school and more	180 (30.72)	75 (39.06)	105 (26.65)	
*Multi*-*deprivation index, n* (*%*)^b^				
Two or more deprivations (Low SES)	106 (18.06)	42 (21.88)	64 (16.20)	0.09
None to one deprivation (High SES)	481 (81.94)	150 (78.13)	331 (83.80)	
*Acculturation* (*mean, SD*)^c^	7.47 (1.5)	7.45 (1.52)	7.49 (1.47)	0.76
*Physical Activity, n* (*%*)^d^				
Moderate/high	408 (70.34)	133 (71.12)	275 (69.97)	0.78
Low	172 (29.66)	54 (28.88)	118 (30.03)	

Bold values indicate statistical significance (*α* = 0.05)

Values are mean ± SD or n (%). Non-overweight/obese = BMI < 25 kg/m^2^; Overweight/obese = BMI ≥ 25 kg/m^2^

^a^Student *t* test for continuous variables (age, acculturation). Chi square test for categorical variables (gender, education, multi-deprivation index, physical activity)

^b^Aggregated number of deprivations based on the sum of the following deprivation indicators: education (none or incomplete primary education), income (household income < US$150 per month) and assets (lowest tertile of possessions weighted asset index) in the same individual

^c^Measured by 10–item acculturation scale. Scores ranged from 0–10 with higher scores indicating higher acculturation

^d^Moderate–to–high PA = at least 600–3000 MET minutes/week. Low PA = less than 150 MET minutes in 1 week

### Sociocultural Factors and Overweight/Obesity

The strongest predictor of overweight/obesity was SES (OR = 0.486; *p* = 0.008). Acculturation was not significantly associated with overweight/obesity in either gender group. No significant interactions were observed between gender and SES or gender and acculturation (*p* = 0.8932 and *p* = 0.1763, respectively). Interaction effects between SES and acculturation were not significant (*p* = 0.67). Holding acculturation, gender, age, and education constant, the odds of being overweight/obese were 51.4 % lower for rural–to–urban migrants with low SES compared to those from higher SES. Similar associations were observed before and after adjusting for PA (Table 2). A trend toward significance was observed when analyses were conducted separately by gender (females OR = 0.535; *p* = 0.07; males OR = 0.444; *p* = 0.06) (results not shown).

**Table 2:** Logistic regression analysis predicting obesity in migrants

	Odds ratio	95 % CI	*χ* ^2^	*p* value
Socioeconomic status^a^	0.486	0.285–0.828	7.0498	**0.0079**
Acculturation^b^	1.087	0.949–1.246	1.4491	0.2287

Bold values indicate statistical significance (*α* = 0.05)

^a^Adjusted for age, gender, education and acculturation

^b^Adjusted for age, gender, education and socioeconomic status

### Structural Equation Modeling

The results showed the data fitted the hypothesized model when acculturation and SES were modeled as correlates (Table 3).

**Table 3:** Summary of fit indices for path models of SES and acculturation

Models	*χ* ^2^	*Df*	P	CFI	TLI	RMSEA	WRMR	∆*χ* ^2^	∆*df*	*p* value
*Acculturation*										
All participants	1.39	4	0.85	1.00	1.44	0.00	0.296			
Multi-group process										
M_1_ no constrains	3.64	6	0.72	1.00	1.75	0.00	0.560			
M_2_ all path loadings constrained	8.14	12	0.77	1.00	1.61	0.00	0.844	4.506	6	0.619
M_3_ all variances constrained	57.04	14	0	0.8	0.57	0.10	1.509	48.891	2	<0.0001
*Socioeconomic status*										
All participants	2.67	4	0.61	1.00	1.22	0.00	0.443			
Multi-group process										
M_1_ no constrains	4.34	6	0.63	1.00	1.53	0.00	0.619			
M_2_ all path loadings constrained	9.04	12	0.70	1.00	1.47	0.00	0.895	4.702	6	0.581
M_3_ All variances constrained	59.03	14	0	0.79	0.55	0.11	1.538	49.988	2	<0.0001

Bold values indicate statistical significance (*α* = 0.05)

RMSEA: <0.05 (good), <0.08 (acceptable); CFI/TLI: > 0.95 (great), >0.93 (better), >0.90 (good)

Table 4 displays the regression coefficients β and standard errors for each significant path. The empirical model manifested the following pathways: (1) SES had a direct effect on overweight/obesity independent of gender. Rural–to–urban migrants with low SES were less likely to be overweight/obese than their wealthier counterparts; (2) PA was not a significant mediator of the relationship between SES and overweight/obesity; and (3) acculturation level was not associated with overweight/obesity in this population.

**Table 4:** Direct and indirect effects of SES and acculturation on obesity

Independent variables	Direct effects	Indirect effects^b^
Socioeconomic status^c^	−0.495 (***p*** **=** **0.002**)	0.003 (*p* = 0.719)
Acculturation^d^	0.048 (*p* = 0.251)	−0.002 (*p* = 0.607)

Dependent variable = overweight/obesity status (overweight/obese = 1 vs. non overweight/obese = 0)

^a^Mediator = Physical activity; ^b^ Low SES = 1; high SES = 0. Adjusted for age, gender, education and acculturation; ^c^ Adjusted for age, gender, education and deprivation index

## Discussion

Contrary to emerging evidence suggesting a higher prevalence of overweight/obesity in groups with the lowest SES, this study showed that, among rural–to–urban migrants, SES was positively associated with their BMI status. Similar associations have been reported in a number studies with non-migrant groups in Peru [41], Brazil [42], Ecuador [43], and Colombia [44].

Acculturation was not associated with overweight/obesity in our sample. An appropriate instrument to measure acculturation among within country rural–to–urban migrants has not been developed. The set of items chosen from two separate instruments developed for and validated among Hispanic immigrants in the United States were believed to have universal applicability. However, the lack of association between acculturation and overweight/obesity may suggest that the unique cultural characteristics of our sample may require a group–specific approach to acculturation. A major challenge for future studies in this area is to capture these unique cultural dimensions when examining the impact of acculturation on overweight/obesity.

A majority of migrants in this study reported moderate–to–high PA levels independent of their gender. In high income countries, immigrants, particularly those with low SES, are more likely to report lower levels of PA [45]. Urban planning, perceived crime, traffic safety, and lack of access to recreational equipment and programs that support an active lifestyle have a considerable impact on their activity patterns [46–48] Our findings suggest that in LMIC, migrants who have moved from rural–to–urban areas seem to lead more active lifestyles probably due to characteristics of their jobs that may require constant physical mobility and the need to be more physically active to fulfill the necessities of everyday living, e.g., walking to different locations. Differences in PA by SES were not explored. However, past research among non-migrant and migrant groups in similar settings has reported an inverse association between SES and PA levels [49]. This further supports the importance of considering the socioeconomic and cultural contexts in which behavioral adoption and practice takes place. Results of the mediation analysis indicate that the direct effect of SES on overweight/obesity was not explained by PA. Whereas this could be partly attributable to the use of self–reported data, contextual factors such as the social and physical environment already discussed need to be considered.

### Strengths and Limitations

Strengths of this study include a representative sample size, the use of a summary measure of acculturation and the development of a deprivation index to assess SES. Compared to other migrant studies, a potential for selection bias was most likely reduced as mass migration from Andean communities such as Ayacucho (rural setting) to Lima (urban setting) was driven by guerrilla violence, political instability, and deepening poverty [50]. A single acculturation score that accounts for different characteristics often clustered within an individual may give a more accurate representation of acculturation than each indicator independently. A deprivation index is considered a more reliable indicator of SES than household income [35].

Limitations include the cross–sectional design that prevents causal interpretation of the relationship between sociocultural factors and overweight/obesity. Because of the limited number of individuals in the obesity category and to maintain sufficient study power, we did not analyze the data for each group separately. The adapted acculturation scale used in this study may not be applicable to our population. Some of the items were scored at different scales for which reliability could not be calculated. The magnitude of the mediation effect may have differed if objective measures of PA were employed. Over–reporting of PA remains an important limitation among studies using self–reported measures of PA and the IPAQ–SF is no exception [51]. Finally, our hypothetical model did not include other possible mediators such as diet, smoking, emotional factors, and lack of sleep.

The results of this study add to the current understanding of the impact of sociocultural dynamics on the global overweight/obesity epidemic but leave many questions unanswered. It appears that efforts toward global overweight/obesity prevention should be tailored to community context, particularly socio–demographic composition, characteristics of the built environment, and previous and current health behavior performance. Given the complexity and interaction of these factors, a “one size fits all” for successful overweight/obesity prevention strategies seems unlikely. For instance, modifications to dietary intake, chronic stress or habitual sleep patterns may be more effective in our population than increasing energy expenditure. In addition, considering that global gender disparities in obesity exist, gender-specific or gender-tailored solutions may be also necessary.

This study is one of very few which has attempted to investigate the potential pathways through which sociocultural factors and overweight/obesity may be linked among migrants from rural-to-urban areas in LMIC. Findings of a positive association between SES and overweight/obesity suggest that mechanisms underlying this relationship may differ depending on the geographic setting (i.e., international migrants from low socioeconomic backgrounds are more likely to be obese in high income countries probably due to high levels of urbanization, easy access to transportation and changes in their dietary habits and the sociocultural context (i.e., changes in cultural beliefs and values around PA and/or diet may be more apparent among international migrants than internal migrants as they are exposed to cultural and social stresses that markedly differ from their own) in which migrants are embedded. The role of acculturation as a risk factor for the development of non–communicable diseases needs to be better understood. Future research should further explore the directional effects of acculturation and overweight/obesity in this population. However, this should be preceded by the development of a culturally valid acculturation scale in order to be fully applicable to internal migrants in LMIC.
